# P-2308. Effect of Antibiotic Therapy on Preventing Subsequent Urinary Tract Infection in Early Kidney Transplant Recipients with Asymptomatic Bacteriuria

**DOI:** 10.1093/ofid/ofae631.2460

**Published:** 2025-01-29

**Authors:** Geonui Kim, Eui Jin Chang, Seongman Bae, Jiwon Jung, Min Jae Kim, Yong Pil Chong, Sang-Oh Lee, Sang-Ho Choi, Sung-Han Kim

**Affiliations:** Asan Medical Center, Songpa-gu, Seoul-t'ukpyolsi, Republic of Korea; Department of Internal Medicine, Asan Medical Center, Seoul, Korea, Seoul, Seoul-t'ukpyolsi, Republic of Korea; Asan Meidical Center, Songpa-gu, Seoul-t'ukpyolsi, Republic of Korea; Asan Medical Center, Songpa-gu, Seoul-t'ukpyolsi, Republic of Korea; Asan Medical Center, Songpa-gu, Seoul-t'ukpyolsi, Republic of Korea; Asan Medical Center, Songpa-gu, Seoul-t'ukpyolsi, Republic of Korea; Asan Medical Center, Songpa-gu, Seoul-t'ukpyolsi, Republic of Korea; Asan Medical Center, Songpa-gu, Seoul-t'ukpyolsi, Republic of Korea; Asan medical center, Seoul, Seoul-t'ukpyolsi, Republic of Korea

## Abstract

**Background:**

The efficacy of treating asymptomatic bacteriuria during the early peri-transplantation period in kidney transplant (KT) recipient has not been properly assessed. In this study, we assessed the effectiveness of treating asymptomatic bacteriuria in first three months after KT and one month before, including bladder irrigation specimen obtained from operating room.

**Figure:**
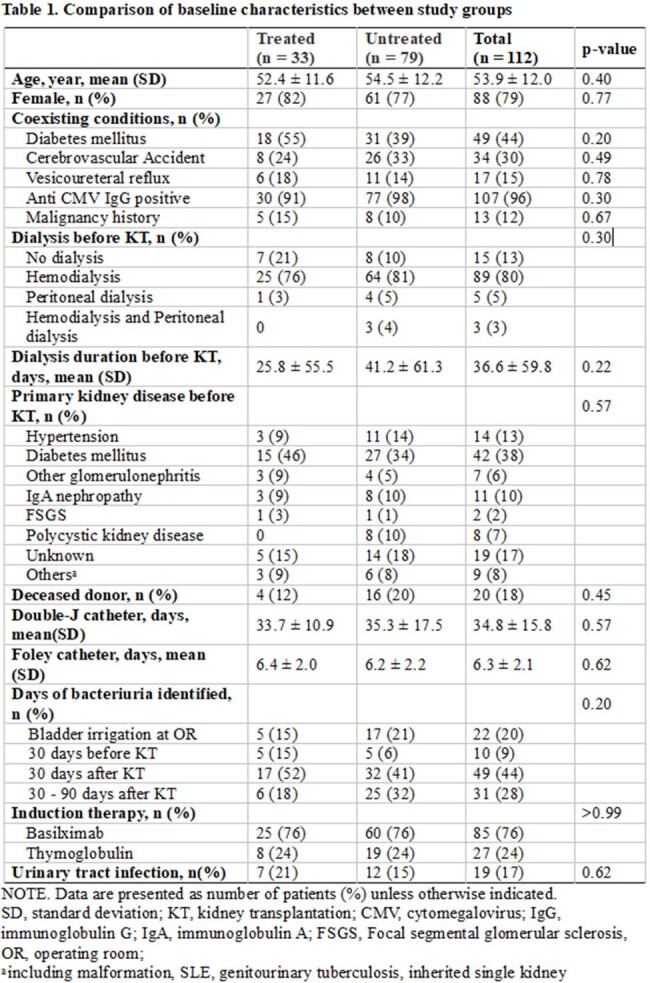

**Methods:**

A single center retrospective cohort study was conducted on patient who underwent KT. KT recipient with asymptomatic bacteriuria within 30 days before and 90 days after the transplantation between January 2020 and January 2023 were included. The frequency of subsequent urinary tract infection (UTI) within 90 days of detecting asymptomatic bacteriuria was compared between antibiotic-treated and untreated groups.

**Figure:**
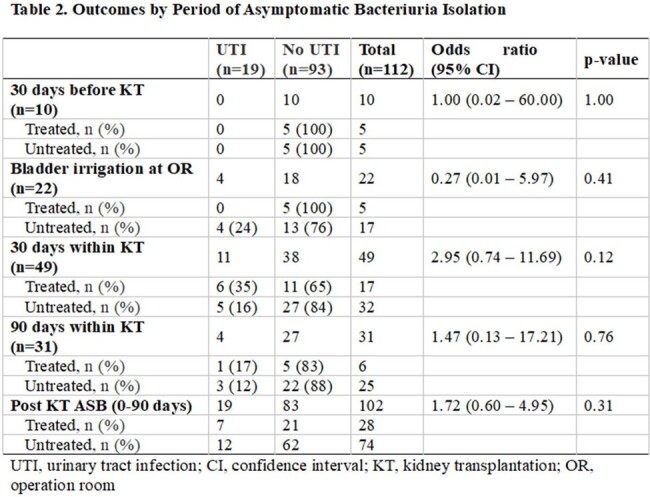

**Results:**

A total of 112 KT recipients with asymptomatic bacteriuria were included; of these, 33 (29 %) received antibiotic treatment while 79 (71 %) did not. Nineteen patients (17%) developed symptomatic UTIs within 90 days of detecting asymptomatic bacteriuria; 21% (7/33) in the antibiotic-treated group and 15% (12/79) in the untreated group (odds ratio, 1.53; 95% confidence interval, 0.53 to 4.24). Untreated asymptomatic bacteriuria was not significantly associated with subsequent UTI in multivariable logistic regression analysis (adjusted odds ratio, 1.32, 95% confidence interval, 0.45 to 3.85; p=0.61) after adjusting gender, vesicoureteral reflux, malignancy, type of donated kidney and induction immunosuppressive drug.

**Figure:**
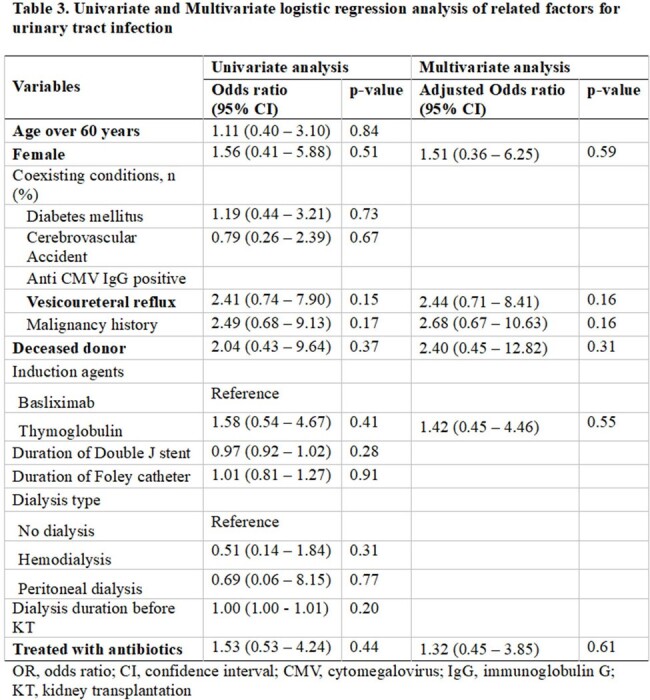

**Conclusion:**

We found that antibiotic therapy for patients with asymptomatic bacteriuria during the early peri-transplantation period did not confera significant advantage in preventing subsequent UTIs. Prescribing antibiotics for asymptomatic bacteriuria in KT recipients should be approached with caution due to the potential risk of colonization or infection by resistant bacteria.

Figure 1.

**Figure:**
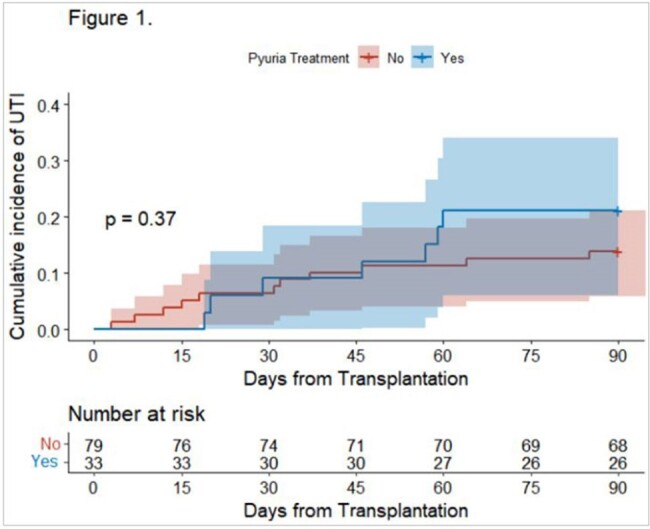

Kaplan-Meier estimates of UTI incidence in patients with asymptomatic bacteriuria after KT ; Log-rank test showed that treated group showed that UTI occurred later than untreated group. (p=0.37)

**Disclosures:**

All Authors: No reported disclosures

